# Population-based screening for celiac disease reveals that the majority of patients are undiagnosed and improve on a gluten-free diet

**DOI:** 10.1038/s41598-022-16705-2

**Published:** 2022-07-25

**Authors:** Jan-Magnus Kvamme, Sveinung Sørbye, Jon Florholmen, Trond S. Halstensen

**Affiliations:** 1 Department of Clinical Medicine, Faculty of Health Sciences, University of Tromsø, 9037 Tromsø, Norway; 2 Department of Gastroenterology, University Hospital of North Norway, 9037 Tromsø, Norway; 3 Department of Pathology, University Hospital of North Norway, 9037 Tromsø, Norway; 4 Institute of Oral Biology, University of Oslo, P.O. Box 1052, 0316 OsloBlindern, Norway; 5 Medical Department, Lovisenberg Diaconal Hospital, Oslo, Norway

**Keywords:** Gastrointestinal diseases, Autoimmune diseases

## Abstract

The impact of a gluten-free diet (GFD) on screen-detected celiac disease (CD) is currently ambiguous. We aimed to identify the population-based prevalence of undiagnosed adult CD and examine the impact of a GFD on screen-detected CD. In total, 12,981 adults participated in a population-based health study in Tromsø, Norway. Participants with increased levels of anti-tissue transglutaminase-2 IgA or anti-deamidated gliadin peptide IgG were invited to undergo gastroduodenoscopy with both histological and immunohistochemical examination of small-bowel biopsies. The prevalence of previously diagnosed CD was 0.37%. Additionally, the prevalence of previously undiagnosed CD was 1.10%. Thus, 1.47% of the population had CD, of whom 75% were previously undiagnosed. A GFD resulted in significant improvements in overall gastrointestinal symptoms, diarrhea, and health-related quality of life, with reduced abdominal discomfort (76%) and improved levels of energy (58%). The large majority of patients with adult CD were undiagnosed and benefited from a GFD with reduced gastrointestinal symptoms and improved health-related quality of life. In clinical practice, there should be a low threshold for CD testing even in the absence of abdominal complaints because most adult patients appear to consider their symptoms a part of their normal state and therefore remain untested and undiagnosed.

**Trial registration:** Clinical Trials. Gov Identifier: NCT01695681.

## Introduction

Celiac disease (CD) is characterized by gluten-induced inflammation of the small intestinal mucosa and presents with a wide range of symptoms^1^. As many patients have vague and nonspecific symptoms, a diagnostic delay is common, and the majority remain undiagnosed^2^.

Most serology-based prevalence studies report that, worldwide, 1–2% of the population has CD, a prevalence that drops to 0.7% if only histopathologically proven CD is included^3^. CD may be diagnosed at all ages^4^. Although the differences in prevalence between serology-screened CD with or without biopsy verification may depend on the diagnostic criteria applied, there may also be regional differences^3^. No population-based prevalence data exist for Norway, but 0.35% of blood donors had undiagnosed CD based on serology followed by biopsy verification^5^, suggesting a lower prevalence than that in the other Nordic countries. However, this prevalence estimate may have been underestimated due to the use of the presumably less sensitive endomysium test and not anti-transglutaminase-2 IgA (TG2-IgA).

Because reduced gluten intake has become more prevalent in the population worldwide^6^, undiagnosed CD may have fewer characteristic mucosal alterations, making a definite histopathological diagnosis more difficult. However, diagnostic accuracy may be improved by the immunohistochemical enumeration of intraepithelial T-cell receptor (TCR) γ/δ+ T cells^7,8^ and the detection of lamina propria IgA-TG2 immune complexes^9^. Screening for CD has mostly been studied in risk groups such as first-degree relatives and patients with autoimmune diseases^10,11^. A gluten-free diet (GFD) reduced gastrointestinal symptoms in relatives of CD patients^12^ and improved low bone density in another study^11^. However, there is a lack of research exploring the impact of a GFD in CD patients detected after screening of the general population.

We therefore aimed to study the population-based prevalence of previously undiagnosed CD and evaluate the impact of a GFD on screen-detected CD.

## Methods

### Study population

The Tromsø study is a population-based study with repeated health surveys of the inhabitants of Tromsø County, Norway, a mixed urban–rural community. The 6th cross-sectional survey was conducted in 2007/2008. A random selection (n = 19,762) representing half of the population aged 30–87 years (n = 39,335) was invited to participate. In total, 6053 men and 6928 women attended, resulting in participation rates of 62.9% and 63.8%, respectively. Serum was available from 12,190 individuals, which represents approximately one-third of the adult population aged > 30 years in the community. The participants were aware that they could be tested for various diseases, but CD was not explicitly stated. The regional board of research ethics (REK-North, Tromsø Norway) approved the whole study, and each participant provided written informed consent before inclusion. All methods were performed in accordance with the guidelines and regulations from the regional board of research ethics.

### Serology and other laboratory parameters

Serum from nonfasting venous blood samples was stored in sealed tubes at − 20 °C for up to 4 years. The samples were analyzed for anti-TG2-IgA (EliA Celikey^®^ IgA, Phadia, Thermo Fisher Scientific, NYSE: TMO) and anti-deamidated gliadin peptides IgG (DGP-IgG) (EliA™ GliadinDP IgG, Phadia, Thermo Fisher Scientific, NYSE: TMO) on the Phadia 250 system (Thermo Scientific, NYSE: TMO) by the Department of Laboratory Medicine, University Hospital, North Norway (accredited clinical laboratory), according to the instructions provided by Phadia (ThermoFisher Scientific, NYSE: TMO). The upper limit of the normal titer was set to < 7 U/mL based on previous studies^13,14^. IgA was measured in all participants, and selective IgA deficiency was defined as serum IgA < 0.07 g/L. Increased serum TG2-IgA or DGP-IgG levels were not controlled with a second sample. The medical laboratory scientist was not informed about any clinical symptoms.

Blood hemoglobin (reference, men 13.0–17.0 g/dL; women 11.5–16.0 g/dL), vitamin B12 (reference 145–569 pmol/L), folate (reference ≥ 10 nmol/L) and ferritin (reference, men 29–383 µg/L; women 10–167 µg/L) were measured using standard laboratory methods in the hospital^15^. All patients undergoing endoscopy were typed for the HLA-risk alleles by the Department of Immunology and Transfusion Medicine, Oslo University Hospital (accredited clinical laboratory, DNA-based genomic sequence-specific oligonucleotide (SSO) typing): HLA-DQA1*05:01-DQB1*02:01 (DQ2.5) and DQA1*03-DQB1*03:02 (DQ8) and if either was negative, DQA1*02:01-DQB1*02:02 (DQ2.2) and DQA1*05:05-DQB1*03:01 (DQ7.5).

### Gastroduodenoscopy with small bowel biopsies

**Figure 1:**
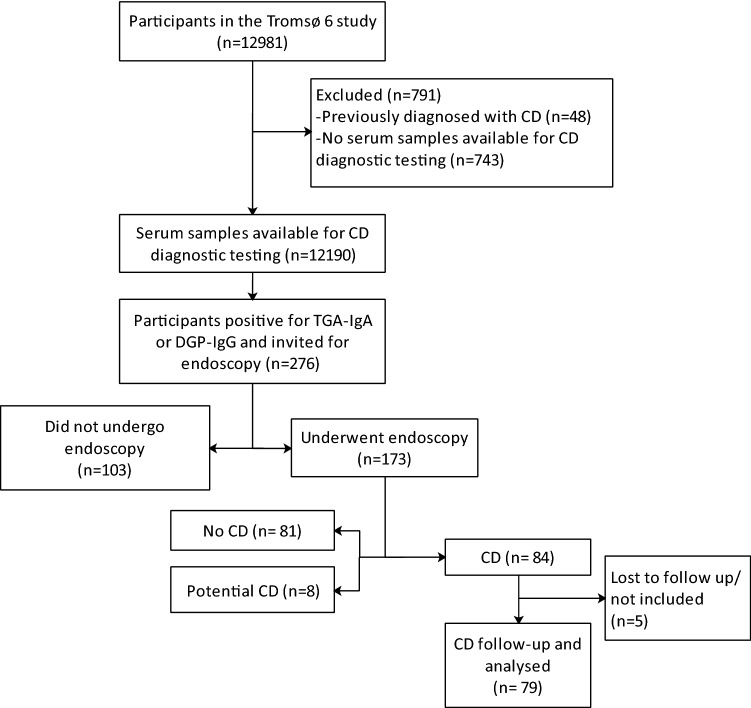
Flowchart of the study. *CD* celiac disease, *TG2-IgA* tissue transglutaminase 2-IgA antibodies, *DGP-IgG* deamidated gliadin peptide-IgG antibodies.

All individuals with increased levels of either TG2-IgA or DGP-IgG and no previous diagnosis of CD (n = 276; Fig. 1) received a letter of invitation recommending gastroduodenoscopy with biopsy for further diagnostic evaluation. The letter included clear advice on not starting a GFD before endoscopy. One written reminder was sent to those who did not respond to the initial invitation. The endoscopic examinations were performed at the Department of Gastroenterology, University Hospital North Norway, Tromsø, in 2013 and 2014.

During gastroduodenoscopy, single-bite biopsy specimens were obtained from the duodenal bulb (n = 4) and the distal part of the duodenum (n = 6)^16,17^. The biopsy samples were divided into two batches: two bulb and four distal duodenal biopsies were formalin-fixed, paraffin-embedded, sectioned (4 µm) and stained with hematoxylin and eosin; and two bulb and two distal duodenal biopsies were fixed in ice-chilled periodate-lysine fixative supplemented with 1% paraformaldehyde (PLP) for 2 h, infiltrated in 20% sucrose at 4 °C for 30 min, oriented on a slice of carrot, embedded in optimal cutting temperature compound (OCT; Tissue-Tek, Miles Laboratories, Elkhart, IN, USA), snap-frozen in liquid nitrogen-cooled isopentane and stored at − 70 °C until cryosectioning at the University of Oslo.

### Multicolor immunohistofluorescence

Cryosections (4 µm) were incubated overnight at 4 °C with the following combinations of unlabeled mAb: the δ chain of the T-cell receptor (TCR) γ/δ complex (Thermo-Fisher Scientific, clone 5A6. E9, IgG1) combined with mAb to CD3 (clone RIV-9, IgG3), as detailed elsewhere^7^. A mAb against human transglutaminase-2 (Neomarker/Abcam, clone CUB7402, IgG1) combined with rabbit antiserum against human IgA (Dako, Denmark) was used to detect the colocalization of IgA and TG2, representing IgA-anti-TG2 immune complexes^18^. The secondary reagents were various combinations of Alexa 488- or 594-conjugated anti-mouse subclass-specific goat antisera in combination with Alexa 488- or Alexa 594-conjugated goat anti-rabbit IgG (all from Molecular Probes, Thermo Fisher Scientific, Waltham, MA, USA). DAPI (4′,6-diamidino-2-phenylindole; Abcam) was occasionally added as a nuclear fluorescence marker to visualize the intraepithelial location of T cells.

### Microscopy, evaluation, and scoring

All routine hematoxylin and eosin-stained sections were examined by the same pathologist (SS) first in a diagnostic blinded manner using the modified Marsh-Oberhuber classification system^19^ with < 25 intraepithelial lymphocytes (IEL)/100 epithelial cells as the upper limit of normal^20^. If the findings were different in the bulb and distal part of the duodenum, the highest degree of pathology was recorded as the result. All immunohistofluorescence-processed sections were also stained with hematoxylin and eosin and evaluated before immunohistofluorescence examination by another observer (TSH) in a diagnostic blinded manner using a Zeiss Axioplan-2 microscope under 250–400 times magnification equipped with single-, double-, and triple-color fluorochrome filter blocks that allowed the individual and simultaneous examination of the green, red and blue emissions (Carl Zeiss, Germany). All or a minimum of 100 CD3+ IELs predominantly in the villous or at the surface epithelium were examined for TCR γ/δ expression in each biopsy sample. The intensity of IgA immunofluorescence in the TG2+ areas (colabeling, representing IgA-TG2 immune complexes) was scored semiquantitatively. CD was defined as an increased serum TG2-IgA titer (≥ 7 U/mL) and/or immunohistochemically detected IgA-TG2 immune complexes in the lamina propria and intestinal crypt cell hyperplasia with epithelial lymphocytosis (> 25 IELs/100 epithelial cells in hematoxylin and eosin-stained duodenal or bulbar sections (Marsh-Oberhuber histological grade 2 or higher)^21^. Immunohistochemical detection of more than 15% TCRγ/δ+ CD3+ IELs in sections with epithelial lymphocytosis and crypt cell hyperplasia^7^ or > 6.5 intraepithelial TCR γ/δ+ CD3+ T cells per 100 epithelial cells were used to discriminate CD from CD mimics^8^. All histological classifications were revaluated by TSH and SS. The term potential CD was used to describe individuals with ≥ 7 U/mL serum DGP-IgG or TG2-IgA titers with or without detectable mucosal IgA-TG2 immune complexes with more than 20 CD3+ intraepithelial CD3+ T cells per 100 epithelial cells^22^ and > 15% intraepithelial TCR γ/δ+ T cells or > 4.5 intraepithelial TCR γ/δ+ CD3+ T cells per 100 epithelial cells^23^ in patients carrying HLA-DQ2.5 or HLA-DQ8 but without villous atrophy (Marsh-Oberhuber grade 0–I lesions)^24^. These participants were not defined as having intestinal CD and therefore not recommended a GFD, but they could also not be included in the non-CD group. The patients diagnosed with CD received GFD instructions from a clinical dietician. Adherence to the diet was evaluated by self-report.

### Assessment of gastrointestinal symptoms and health-related quality of life

Gastrointestinal symptoms and health-related quality of life (HRQOL) were assessed using two self-administered, structured questionnaires widely used in CD studies^16,25^. The participants were advised to rate each item on the questionnaires as it had applied to them during the preceding week. The validated Norwegian version of the Gastrointestinal Symptoms Rating Scale (GSRS) (Olafsson, Haukeland University Hospital, Bergen, Norway)^26^ includes 15 questions exploring the five abdominal symptom dimensions: diarrhea, reflux, indigestion, abdominal pain, and constipation^27^. Each question is scored on a seven-point Likert scale, where a score of 1 represents no symptoms, and a score of 7 represents very serious symptoms. The mean score for each dimension and the total score were compared between patient groups. A lower score indicates fewer gastrointestinal symptoms. The validated Norwegian version of the Psychological General Well-being (PGWB) scale (Helvik, St. Olavs Hospital, Trondheim, Norway)^28,29^ consists of 22 items, each of which is scored on a six-point Likert scale, describing six nonoverlapping dimensions: anxiety, depressed mood, positive well-being, self-control, vitality, and general health. An overall index is calculated as the sum of the responses to each dimension. A higher score indicates better health-related quality of life. The patients newly diagnosed with CD were assessed with the GSRS and PGWB scale twice: first, during the endoscopy visit (baseline), the results were compared with those of the participants without CD in a case–control design and then at follow-up after adhering to a GFD (mean: 16 months, range: 12–22 months).

### Self-reported changes in symptoms

After pilot testing by clinically experienced researchers, two questions were included to examine changes in symptoms on a GFD. (1) Regarding abdominal complaints, how are these after changing your diet? (Abdominal complaints included feeling unwell, experiencing borborygmus, and experiencing changes in bowel habits/diarrhea.), (2) How have your energy and feeling of fitness changed after changing your diet? Both questions were answered on a seven-point Likert scale, from “very much worse” to “very much better”.

### Statistical analyses

Differences between groups at baseline were compared using independent sample *t* test, the Mann–Whitney *U* test, or the chi-squared test, as appropriate. The changes in scores after adopting a GFD were compared using paired-samples *t* tests. The preceding statistical tests were performed using SPSS version 26 for PC (SPSS, Inc., Chicago, IL, USA). Correlations between TG2-IgA and DGP-IgG serum levels were tested using the nonparametric Spearman’s correlation coefficient using GraphPad Prism 9.1.0. A two-sided p value < 0.05 was considered significant.

### Ethics approval and consent to participate

The regional board of research ethics (REK-North, Tromsø Norway) approved the study (28.11.2011), and each participant provided written informed consent before inclusion. The study has been registered in Clinical Trials. Gov with identifier number NCT01695681 (date of first registration 28.09.2012).

## Results

### Prevalence of celiac disease

Information from the primary self-administered questionnaire revealed that 90 individuals reported being on a GFD, of which 42 were due to known CD, while 46 did not have CD, and two did not know. Similarly, 218 individuals reported having “been previously examined by upper endoscopy and diagnosed with CD”. This self-reported information concerning CD diagnosis was validated using the corresponding medical records and histology reports. CD was defined as Marsh-Oberhuber grade 2 or 3 lesions. The diagnosis of CD was confirmed in 48 individuals (23 men and 25 women). Thus, 0.37% (48/12,981, 95% CI 0.36–0.38) of the participants had previously been diagnosed with CD.

Serum samples were missing from 743 individuals, leaving 12,190 participants available for serological screening. In total, 2.26% (276/12,190) of these participants were positive (≥ 7 U/mL) on one of the serological tests, and 53% of those participants (145/276) had increased TG2-IgA titers. The proportion of men who were positive on one of the serological tests was 2.28% (129/5667), similar to the proportion in women, 2.25% (147/6523) (p = 0.90). Furthermore, of the 233 individuals who had increased serum DGP-IgG titers, only 44% (102/233) had an increased TG2-IgA titer. Thus, of the 12,190 participants, 1.19% (145/12,190) had increased TG2-IgA titers, 1.91% (233/12,190) had increased DGP-IgG titers, and 0.84% (102/12,190) had increased TG2-IgA and DGP-IgG titers.

**Figure 2:**
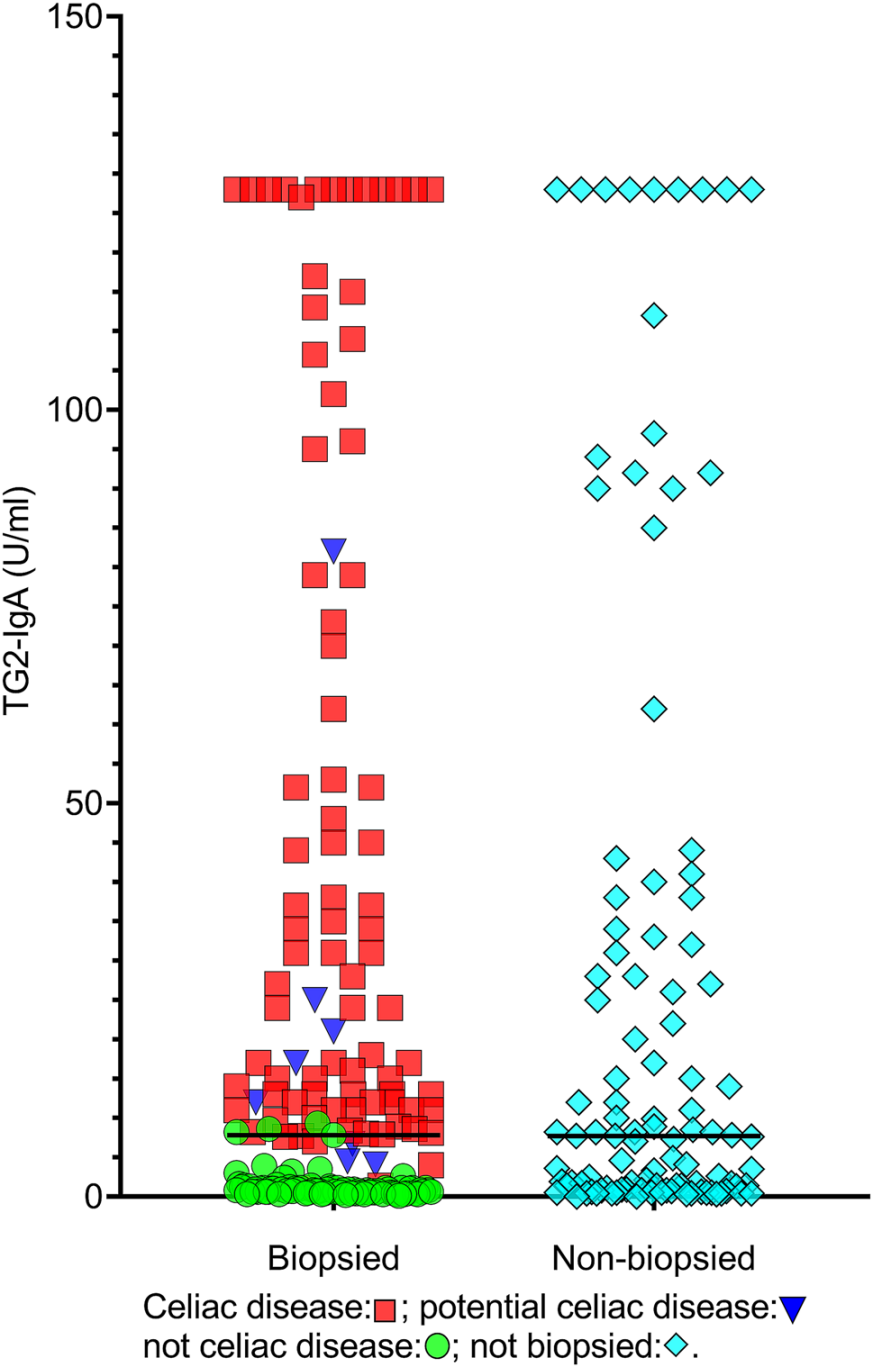
Serum TG2-IgA titers in participants with and without a biopsy-verified diagnosis. Serum TG2-IgA levels in participants with a biopsy-verified diagnosis (left panel, n = 173) had a similar distribution to those in participants who were not biopsied (right panel, n = 103, light-blue rhombi). The horizontal line indicates median TG2-IgA titers (U/mL). *TG2-IgA* tissue transglutaminase 2-IgA antibodies.

After initial and repeated invitations, 63% (n = 173, mean age 65.5 years) of the 276 serology-positive participants (women, 66%; men, 59%; p = 0.25) underwent gastroduodenoscopy (Fig. 1). All of the attending participants reported consuming gluten. The nonattending participants were older (mean 69.3 years, p < 0.05) but had similar serum TG2-IgA levels (Fig. 2) and sex distributions (not shown).

CD was confirmed by biopsy in 84 individuals (49%), of whom 81 (96%) had Marsh-Oberhuber grade 3a–c lesions and three had grade 2 lesions (4%). See Supplementary material, S4 for details about IEL/100 epithelial cells, HLA-DQ and serology. Immunohistochemical examination confirmed the diagnosis in 19 cases, which included the identification of 8 cases with potential CD. Supplementary material, S1 for details. All participants with CD carried either HLA-DQ2.5 (87%), DQ8 (11%), DQ2.2 (13%), or DQ7.5 (13%).

The number of undiagnosed CD in the serum-positive but unbiopsied participants (n = 103) was estimated to be 50, both by using the TG2-IgA titer-associated disease prevalence and the disease prevalence in the biopsied population, as the TG2-IgA titer distribution was almost identical (Fig. 2) in the two groups (see Supplementary material S2 for details). Thus, 49% of these 103 unbiopsied participants may have had CD (n = 50).

In total, 134 (84 + 50) of the 12,190 participants had previously undiagnosed CD (1.10%, 95% CI 0.91–1.27). Adding this prevalence (1.10%) to that of previously diagnosed patients (0.37% of 12,981 participants), 1.47% (95% CI 1.26–1.66) of the population had CD, and 75% of these patients were previously undiagnosed.

Comparing the newly diagnosed participants with those without CD (Table 1), there were no significant differences in age, sex, body mass index, family history of CD or hemoglobin level. The latter was not different when stratified by sex, in contrast to folate and ferritin levels, which were significantly lower in men but not in women with CD (not shown). However, all mean levels were within the sex-specific normal references.

**Table 1:** Baseline characteristics in biopsied participants with and without celiac disease.

Characteristic	CD (n = 84)	No CD (n = 81)	p value
Age, mean (SD)	59.8 (10.4)	57.9 (11.1)	0.27^a^
Male sex, % (n)	56.0 (47)	40.7(33)	0.05^b^
Family history of CD, % (n)	16.9 (14)	14.8 (12)	0.75^b^
BMI, mean (SD), kg/m^2^	26.3 (4.8)	27.0 (5.0)	0.39^a^
**TG2-IgA titer (U/mL)**			
Median (IQR)	29.5 (12.0–91.0)	0.8 (0.5–1.1)	< 0.01^c^
Range	1.1–128	0.1–9.3	
**DGP-IgG titer (U/mL)**			
Median (IQR)	14 (5.8–41.3)	9.9 (7.8–14)	0.06^c^
Range	0.1–185	0.2–137.0	
HLA DQ 2.5^d,e^	71(84.5%)	24 (29.6%)	< 0.01^b^
HLA DQ 2.2	1 (1.2%)	6 (7.4%)	–
HLA DQ 8	9 (10.7%)	17 (21.0%)	< 0.01^b^
HLA DQ 7.5	1 (1.3%)	4 (4.9%)	–
HLA DQ XX	0	29 (35.8%)	–
Hemoglobin g/dL (SD)	14.0 (2.0)	14.0 (0.9)	0.93^a^
Vitamin B12, pmol/L (SD)	361.8 (118.8)	393.8 (170.5)	0.17^a^
Serum folate^f^, nmol/L (SD)	16.3 (6.6)	19.7 (6.9)	< 0.01^a^
Ferritin^f^, µg/L (SD)	132.1 (101.9)	157.2 (142.0)	0.20^a^

p values between groups according to the *t* test^a^, χ^2^ test^b^ or Mann–Whitney *U* test^c^.

^d^No significant difference between men and women within either of the HLA categories (CD p = 0.38, No-CD p = 0.12).

^e^HLA data missing in two CD participants and one No-CD participant.

^f^Only the mean folate level was significantly lower in participants with CD (men and women combined). When stratified for sex, the difference for ferritin reached additional statistical significance in men (p < 0.05) but not in women.

*CD* celiac disease, *No CD* not celiac disease, *SD* standard deviation, *n* number of participants.

### Performance of the serological tests

**Figure 3:**
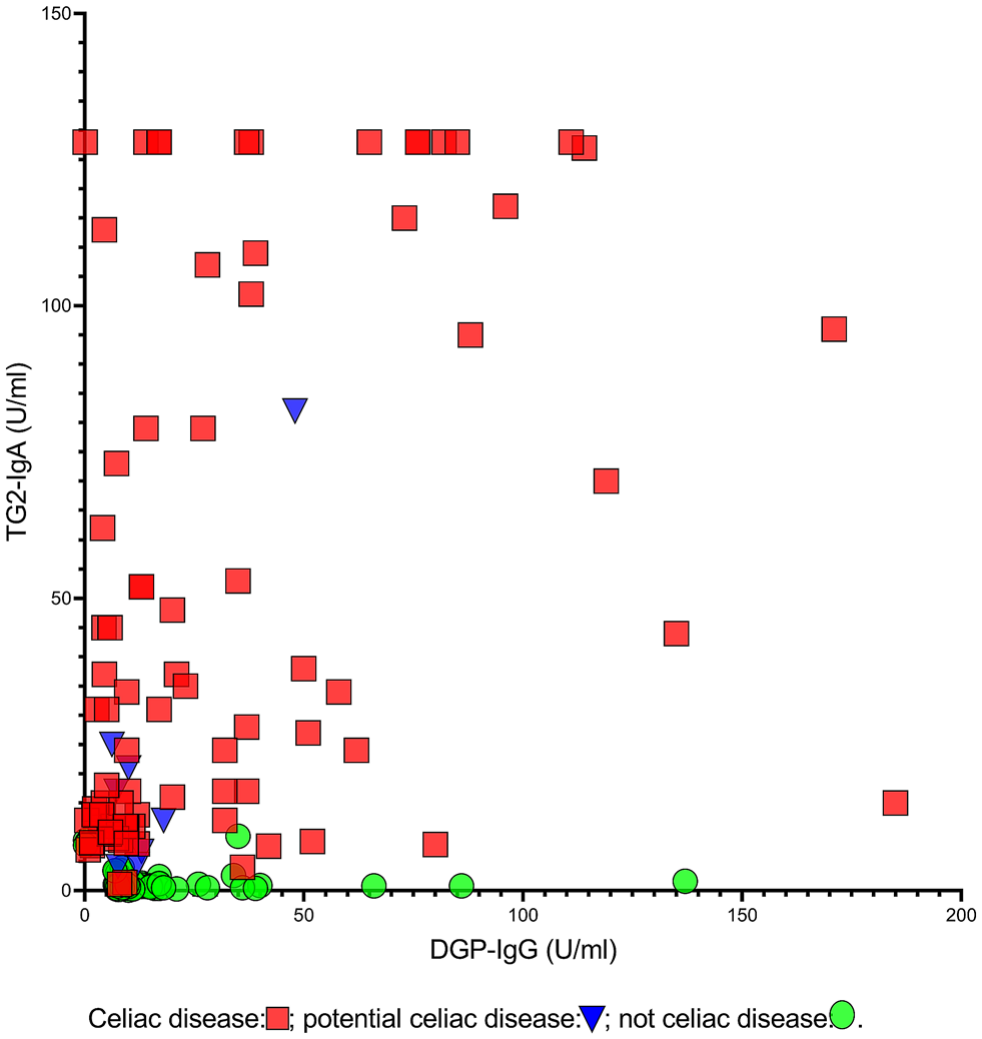
Distribution of serum DGP-IgG and TG2-IgA according to celiac disease status. Serum DGP-IgG (x-axis) and TG2-IgA (y-axis) from the participants (n = 173) with a biopsy-verified diagnosis. Although the DGP-IgG and TG2-IgA titers were significantly correlated in CD patients (p < 0.001, Spearman r = 0.45), they were not correlated in participants without CD. *DGP-IgG* deamidated gliadin peptide-IgG antibodies, *TG2-IgA* tissue transglutaminase 2-IgA antibodies, *n* number of participants.

Although the serum TG2-IgA and DGP-IgG titers (Fig. 3) correlated significantly in patients with CD (Spearman p < 0.001), there were large variations (r = 0.45). The two titers correlated neither in the patients without celiac disease nor in the 103 undiagnosed, unbiopsied participants.

Among the 90 individuals with increased TG2-IgA titers (Table 2), 90% had biopsy-confirmed CD (n = 81), five had potential CD, and four did not have CD. The TG2-IgA and DGP-IgG titers were both increased in 63 of the biopsied participants (36%), of whom most had either CD (n = 58; 92%) or potential CD (n = 4; 6.3%). One HLA-DQ 2.5+ participant with increased serum TG2-IgA (9.2 U/mL) and DGP-IgG (35 U/mL) had normal histology. The DGP-IgG titer was increased, and the TG2-IgA titer was normal in 83 of the 173 participants (48%), of whom three had biopsy-confirmed CD and three had potential CD (3.6%). The remaining participants were considered normal (n = 77, 92%), including two participants with IgA deficiencies (< 0.07 g/L). Although the study was not designed to estimate the positive and negative predictive values of the serology tests (PPV and NPV, respectively), the results showed that an increased TG2-IgA titer predicted CD much better than an increased DGP-IgG titer did (PPV = 0.90 and 0.42, respectively; Table 2). Similarly, 92% of the biopsied participants with normal TG2-IgA titers (< 7 U/mL) did not have CD (NPV = 0.92). Having both TG2-IgA and DGP-IgG titers increased was slightly better at predicting CD (PPV = 0.92).

**Table 2:** Distribution of the serological test results among the biopsied participants (n = 173).

Serological test	Celiac disease status, n			Sum	PPV CD	NPV^a^ CD
	CD	Pot. CD	No CD			
**Based on one serological test only**						
TG2-IgA+	81	5	4	90	0.90	
DGP-IgG+	62	7	78	147	0.42	
TG2-IgA^neg^	4	3	75	82		0.95
**Based on two serological tests**						
TG2-IgA+, DGP-IgG+	58	4	1	63	0.92	
TG2-IgA+, DGP-IgG^neg^	23	1	3	27	0.85	
TG2-IgA^neg^, DGP-IgG+	3	3	77	83	0.04	

^a^Potential CD patients were included in the group without CD to calculate the NPV.

*CD* celiac disease, *Pot. CD* potential celiac disease, *No CD* not celiac disease, *PPV* positive predictive value, *NPV* negative predictive value, *TG2-IgA* tissue transglutaminase 2-IgA, *DGP-IgG* deamidated gliadin peptide-IgG, *n* number of patients.

If the serum TG2-IgA and DGP-IgG titers were used to diagnose adult CD without biopsy verification, similar to what has been adapted for children^30^, then most of the highly TG2-IgA-positive patients (> 70 U/mL, PPV = 0.96) would have been correctly diagnosed with CD (see Supplementary material S3 for more details). All 75 participants with TG2-IgA titers ≥ 10 U/mL, regardless of their DGP-IgG titers, had either active (n = 70; PPV = 0.93) or potential (n = 5) CD.

### Impact of a gluten-free diet

At baseline, no significant differences were found in any of the health-related quality of life dimensions between the individuals with screen-detected CD and those without CD (Table 3). However, the individuals without CD reported more abdominal pain and constipation and a higher total GSRS total score than those with CD.

**Table 3:** Gastrointestinal symptoms (GSRS) and health-related quality of life (PGWB) at diagnosis in individuals with (CD) and without (No CD) biopsy-proven celiac disease.

Variable	Mean score (SD)		p value^c^
	CD (n = 84)	No CD (n = 81)	
**GSRS**^**a**^			
Total score	1.77 (0.70)	2.05 (0.72)	0.01
Diarrhea	1.85 (1.18)	2.11 (1.23)	0.17
Reflux	1.38 (0.61)	1.54 (0.83)	0.14
Indigestion	2.15 (0.99)	2.37 (1.01)	0.15
Abdominal pain	1.82 (0.81)	2.29 (0.91)	< 0.01
Constipation	1.65 (0.78)	1.93 (0.88)	0.04
**PGWB**^**b**^			
PGWB index	86.05 (13.99)	83.39 (12.93)	0.21
Anxiety	21.32 (3.30)	20.78 (3.28)	0.29
Depressed mood	13.73 (1.86)	13.53 (1.67)	0.48
Positive well-being	13.19 (2.78)	13.28 (2.47)	0.82
Self-control	13.63 (1.78)	13.40 (1.80)	0.37
Vitality	13.52 (3.78)	12.80 (3.72)	0.22
General health	10.65 (3.14)	9.69 (3.04)	0.05

^a^GSRS: A lower score is better.

^b^PGWG: a higher score is better.

^c^Student’s *t* test (the nonparametric Mann–Whitney *U* test revealed similar results).

The newly diagnosed patients with CD (n = 84) were advised to adhere to a GFD. Five of these patients did not complete the follow-up study for various reasons: one patient died from an unrelated condition; three patients declined to continue a GFD because of lack of motivation; and one patient who was later reclassified from normal to CD after immunohistochemical examination was not included in the follow-up study.

**Figure 4:**
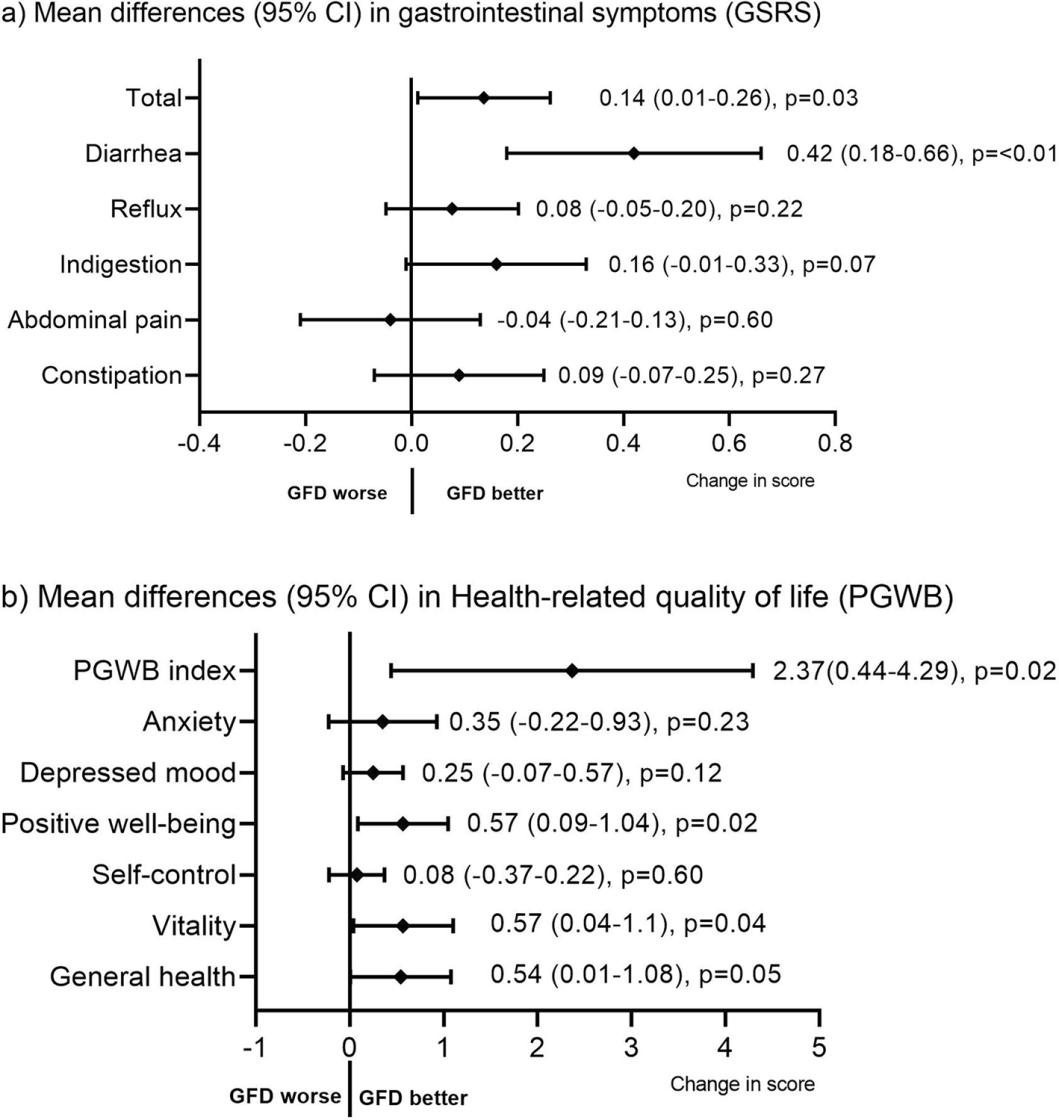
Differences in gastrointestinal symptoms and health-related quality of life on a GFD. Score differences in individuals with a biopsy-proven CD (n = 79) between inclusion and follow-up on a GFD. The direction of change in the GSRS has been reversed. *GSRS* Gastrointestinal Symptoms Rating Scale, *PGWB* Psychological General Well-Being.

**Figure 5:**
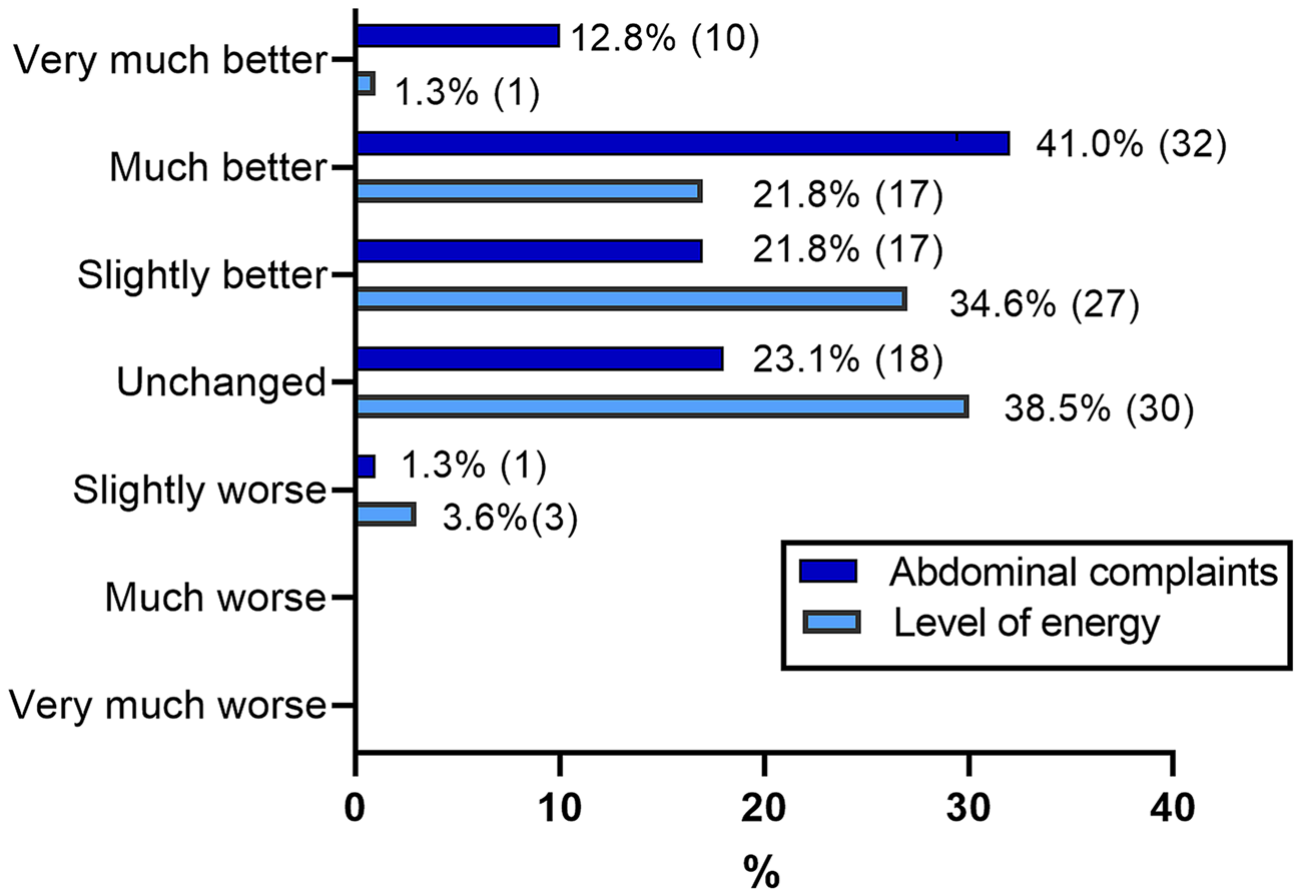
Self-reported changes (%) in symptoms at follow-up on a GFD. n = 78 (one missing), p < 0.05 for the model (χ^2^ test).

After adhering to a GFD, the patients with CD showed improvements in the GSRS total score (p = 0.03) and diarrhea subscore (p < 0.01) (Fig. 4a). The PGWB total score (p = 0.02), vitality (p = 0.04) and positive well-being (p = 0.02) subscores also improved (Fig. 4b). Retrospectively, most patients with CD on a GFD diet reported less abdominal discomfort (76%) and higher levels of energy (58%); however, 23% had no changes in abdominal complaints, and 39% did not experience any change in energy levels (Fig. 5). At follow-up, all patients reported adhering to a GFD, which was reflected in reduced TG2-IgA titers in all except two patients, and the majority (79%) had an almost normalized titer (< 10 U/mL).

### Complications and adverse effects

After gastroduodenoscopy, one patient was hospitalized overnight because of procedure-related gastrointestinal bleeding. She received a blood transfusion but had no ongoing bleeding on endoscopy. At follow-up, one patient reported slightly more abdominal complaints. Four patients reported slightly less energy at follow-up, but three of them also reported significantly reduced abdominal complaints.

## Discussion

The prevalence of adult CD was 1.47% in this large population-based screening study, and the majority (75%) were previously undiagnosed. Adhering to a GFD for more than 1 year reduced gastrointestinal symptoms, particularly diarrhea, and improved the health-related quality of life in these screen-detected patients with CD.

Although the current prevalence of biopsy and immunohistochemically confirmed CD was similar to what has been estimated globally^3^, the high prevalence of undiagnosed disease (1.1%) in this Norwegian population with its well-organized health care system was rather surprising. The prevalence of CD varies across countries and regions due to differences in genetic constitution and gluten intake^31^. The current prevalence was lower than that reported for Finland (2.6%)^32^ but higher than those in several other Northern European countries^3^.

Both the GSRS and PGWB explore a wide range of symptoms and are recommended patient-reported outcome instruments in CD studies^16^. Adhering to a GFD reduced the GSRS total score and diarrhea subscore in the current screen-detected CD patients, similar to the effect in relatives of CD patients with screen-detected CD in Finland^12^. However, whereas the health-related quality of life (PGWB) was improved across several subscores in the current patients, only the anxiety subscore improved with a GFD in screen-detected Finnish relatives^12^. The explanation for this discrepancy is rather obscure, but differences in culture, symptom awareness, and general health anxiety may all have influenced the self-reported scoring. Improved quality of life on a GFD was also observed in clinically diagnosed CD patients in a study from Nachmann et al.^33^, who later observed an association between treatment noncompliance and deterioration of quality of life in these patients^34^.

Despite the availability of adequate serum markers, the proportion of individuals with undiagnosed CD remains high, as illustrated in the current study, in which the majority of cases were detected by the screening. Such a high prevalence of undiagnosed CD appears to be the rule, as several studies have reported similar diagnosed/undiagnosed ratios (1:3–1:5)^32,35^. The difficulties in identifying these undiagnosed patients clinically may rely on their uncharacteristic symptoms with fewer abdominal complaints and lower GSRS total scores than those without CD, even though 96% of the patients had Marsh-Oberhuber grade 3a-c intestinal lesions. Similar results were reported from Olmsted County in Minnesota, where individuals with undiagnosed CD had less diarrhea and dyspepsia than control individuals^36^, as also noted in a Danish serology-based screening study^37^. Another screening study from North America could not identify any single gastrointestinal symptoms that would predict CD^38^. Thus, CD-associated abdominal symptoms may be too vague to be used as disease hallmarks in clinical practice. This observation was also noted by van der Windt et al.^39^ and may partly explain why the majority of adult patients remain undiagnosed.

Immunohistochemistry was included in the present study as an additional diagnostic tool. This aspect resulted in the reclassification of 11 participants and the identification of eight individuals with potential CD. These latter individuals with potential CD were not included in the CD group but could not be considered nonceliac controls. Epithelial lymphocytosis without crypt cell hyperplasia is considered nonspecific and not diagnostic for CD. However, if patients have increased serum TG2-IgA titers and increased density of TCRγ/δ+ intraepithelial T cells^7,8^, with or without detectable submucosal IgA-TG2 immune complexes, they would be classified as having CD in which the degree of intestinal villous atrophy would depend on the amount of gluten consumed. However, as the current guidelines do not include Marsh-Oberhuber grade 1 lesions as CD specific, these lesions were considered potential CD. The clinical impact of a GFD in potential CD remains unknown, as none of the current guidelines recommend a GFD for these subjects.

In the present study, changes in health status were measured both prospectively and retrospectively. Each of these methods is associated with bias. Many patients with CD are not conscious of their symptoms prior to their diagnosis and may become fully aware of their disease burden only after adhering to a GFD for some time^40,41^. Thus, the symptoms at baseline may have been underreported, resulting in an underestimation of the GFD-induced symptom improvement at follow-up. This response shift is a known bias in prospective evaluations and may partly explain why patients reported better symptom improvement in the retrospective than in the prospective evaluation, despite the known recall bias in retrospective evaluation^42^.

The most common reason for dietary failure and suspicion of refractory CD is poor dietary compliance^43^, which is associated with a younger age at diagnosis and a lack of acute gluten-induced symptoms^44^. In the present study, the newly diagnosed patients with CD received dietary instructions from a clinical dietician, and all patients claimed to adhere to a GFD at follow-up. Consequently, the TG2-IgA titers in most of the patients had decreased to less than 10 U/mL after more than 1 year on a GFD.

There were no significant sex differences in the proportions of patients with positive serological test results for CD, biopsy-confirmed CD, or previously diagnosed CD in the present study. The significant female predominance often reported in clinically diagnosed CD prevalence studies^45^ may, therefore, partly be due to gender differences in health behavior. Men are less likely to seek medical attention^35^ and, therefore, are more likely to remain undiagnosed. The sex distribution in this study was similar to that reported from Olmsted County, MN, USA^46^. However, in a large metanalysis by Singh, a slightly higher CD prevalence was found in women than in men^3^.

TG2-IgA and DGP-IgG serum titers are often used as serological markers for CD, as the latter may identify CD patients with IgA deficiency. However, none of the three CD patients detected solely on increased DGP-IgG titers had IgA deficiency. Whereas TG2-IgA directly identifies CD-associated autoantibodies, DGP-IgG indirectly identifies CD-associated increased T-cell reactivity to deamidated gliadin peptides. Gliadin deamidation may also occur during bread baking (heating) and gastric acid treatment, which may explain why increased serum DGP-IgG levels were less sensitive and specific than increased TG2-IgA serum levels in predicting CD. This finding was later noted in both children and adults after the current study was finalized^47^. Although the HLA distribution in the present celiac patients was similar to that reported in most other studies, as the vast majority expressed HLA-DQ2.5 and/or HLA-DQ8^48^, there were some peculiarities. Whereas only 19.33% of the Norwegian population carries the HLA-DQB1*0201, and 13.95% have the HLA-DQ*0302 genes^49^, 29.6% of the participants without CD carried HLA-DQB1*0201 in combination with HLA-DQA1*05:01 (DQ2.5), and 21% carried the HLA-DQ*0302 genes (HLA-DQ8). These findings suggest that these CD-associated HLA-DQ alleles are associated with a gluten-induced immune reaction in nonceliac patients as well, as all patients were selected by having increased GDP-IgG or TG2-IgA serum levels. However, we cannot exclude the possibility that there is a higher prevalence of DQB1*02:01 (HLA-DQ2.5) and/or HLA-DQ*0302 (HLA-DQ8) in northern Norway.

The current official guidelines^35^ recommend that adult CD should be diagnosed with biopsy verification and not based on serology alone. However, it has recently been discussed by Husby et al. whether adult CD may be diagnosed based on serology alone (TG2-IgA > × 10 upper normal limit), similar to childhood CD^50^. Based on the data from the present study, nonbiopsy diagnosis may be considered only in HLA-DQ2.5/DQ8-positive individuals with both TG2-IgA and DGP-IgG serum titers above 10 times the upper normal limit (≥ 70 U/mL), as it otherwise may include patients with potential CD. Biopsy verification remains, therefore, the diagnostic gold standard for the majority of adult individuals with suspected CD.

A major strength of the present study is the population-based design with a large sample size of more than 12,000 participants, accounting for almost 1/3 of the adult population in the local area. To our knowledge, this study is the largest published screening study of CD with biopsy-confirmed diagnoses and dietary intervention thus far and the only one that has included newer diagnostic immunohistopathological techniques, such as the enumeration of intraepithelial TCRγ/δ+ T cells and the detection of mucosal TG2-IgA immune complexes, to validate the diagnosis to improve the diagnostic accuracy^8^. Interestingly, the current, biopsy-verified prevalence is equivalent to serum-based screening studies but higher than those using routine histopathological verification only. This finding illustrates how difficult it may be to discriminate CD mucosa from its mimics^8^ without immunohistochemical examinations.

The present study has some limitations. Examining the impact of a GFD on screen-detected CD without an appropriate control group on a placebo diet containing gluten may be a weakness. Although such a design was used in patients without CD in a short 6-week study^51^, using this strategy for the longer time span (> 1 year) needed to explore the impact of a GFD on patients with screen-detected CD would be difficult.

Another concern is the possible reduction in gluten intake before attending the gastroduodenoscopy. Although all the participants received written information with clear advice on not starting a GFD before gastroduodenoscopy, we cannot exclude the possibility that some CD patients had reduced their gluten intake over time and thereby induced partial remission. However, the immunohistochemical examination would, at least, have identified the partly healed mucosa on a low-gluten diet as potential CD, since treated and potential CD share the characteristic increase in TCRγ/δ+ IEL^7^.

Although many patients may experience an initial “honeymoon effect” after changing to a GFD^52^, adult patients often need a longer time to clinically recover due to slower healing of the intestinal mucosa than previously assumed^53^. Thus, the mean 16-month period the current patients used a GFD prior to reporting its effect may have been too short a time to fully recover from the CD, thus underestimating the benefit of a GFD in these patients.

There is an ongoing discussion about screening for CD^41,54^. Several guidelines recommend screening in patients with gastrointestinal symptoms (e.g., those with irritable bowel syndrome symptoms) and in higher-risk groups, such as patients with insulin-dependent diabetes mellitus and CD family members^55,56^. However, although CD fulfills most of the screening criteria set by the World Health Organization^55^, population-based screening is not recommended by any of the current guidelines. There is both a lack of generally accepted screening criteria and scientific doubts about whether a GFD would improve what is often considered asymptomatic screen-detected CD^35,57^. Moreover, a GFD may be challenging to follow for individuals with vague symptoms. However, the current study revealed that 1.1% of the adult population had undiagnosed CD and, more importantly, that the majority of these screen-detected celiac patients improved on a GFD.

## Conclusions

This large population-based and immunohistopathological verified screening study revealed that 1.47% of the adult population had CD, of which 75% had undiagnosed disease. A GFD reduced symptom burden and improved quality of life in most of these screen-detected patients. Screening of either high-risk groups or individuals with abdominal symptoms may not be sufficient to identify the majority of patients. Thus, screening of the general population may be the only way to identify the majority of untreated CD patients. In clinical practice, there should be a low threshold for CD testing even in the absence of abdominal complaints because the large majority of adult patients consider their symptoms a part of their normal state and therefore remain untested and undiagnosed.

## Supplementary Information

Supplementary Information.

## Data Availability

Data may be available on reasonable request (e-mail: jan-magnus.kvamme@uit.no).

## Abbreviations

CD: Celiac disease
Pot. CD: Potential celiac disease
No CD: Without celiac disease
GFD: Gluten-free diet
PPV: Positive predictive value
NPV: Negative predictive value
TG2-IgA: Tissue transglutaminase 2-IgA
DGP-IgG: Deamidated gliadin peptide-IgG
GSRS: Gastrointestinal Symptoms Rating Scale
PGWB: Psychological General Well-Being
