# Supplementary material for: Population-based screening for celiac disease reveals that the majority of patients are undiagnosed and improve on a gluten-free diet
Source: Sci Rep. 2022 Jul 25;12:12647. doi: 10.1038/s41598-022-16705-2 (PMC9314380; doi:10.1038/s41598-022-16705-2)
Supplement: Supplementary file 1 — Supplementary Information. [file 41598_2022_16705_MOESM1_ESM.docx]

**Supplementary material**

**Supplementary material S1**

**Detailed results from immunohistochemical examination of the biopsies**

As an additional diagnostic tool, immunohistochemistry was included in the present study. Five patients were reclassified from CD to the control group due to difficulties in interpreting the bulbar biopsies, as the accompanying duodenal biopsies did not have intestinal lymphocytosis or increased intraepithelial TCRγ/δ+ T cell densities. Five additional participants with intestinal lymphocytosis (Mars-Oberhuber grade 1 lesion) without increased intraepithelial TCRγ/δ+ T cell densities (median <1 TCRγ/δ+ T cells pr. 100 epithelial cells, range 0.27-2.6) who had increased serum DGP-IgG (mean, 12; range, 7.3-18 U/ml) but not TG2-IgA titers (<1.0 U/ml), were classified as controls.

Eight HLA-DQ2.5- and/or DQ8-positive participants who had increased DGP-IgG (n=7, median, 11 U/ml; range, 6.2-48) and (n=7)/or (n=1) increased TG2-IgA on inclusion (median, 14.5 U/ml; range, 4.1-82 U/ml) or on the day of attending gastroduodenoscopy (median, 7 U/ml; range, 0.6-73 U/ml), were classified as potential CD as the duodenal cryosection revealed >20 intraepithelial CD3+ T cells pr. 100 epithelial cells (median, 31; range, 21-38; Mars-Oberhuber grade 0-1 lesion) and >4.5 TCRγ/δ+ intraepithelial T cells pr. 100 epithelial cells (median, 9; range, 6-16) (Table 4). Six of these participants had additional mucosal IgA-TG2 immune complexes (1+) but no crypt cell hyperplasia.

One patient was reclassified from control to CD due to epithelial lymphocytosis with increased TCRγ/δ+ T cell density, submucosal IgA-TG2 immune complexes, and crypt cell hyperplasia (Mars-Oberhuber grade 2) in the duodenal cryosection. Only one CD patient did not have increased intraepithelial TCRγ/δ+ T cell density. This patient had a high serum TG2-IgA titer (128 U/ml), submucosal IgA-TG2 immune complexes (3+), severe intraepithelial lymphocytosis (51 intraepithelial T cells pr. 100 epithelial cells) and duodenal Mars-Oberhuber grade 3b lesion, but only 6% intraepithelial TCRγ/δ+ T cells (3.06 TCRγ/δ+ pr. 100 epithelial cells).

**Table 4. Detailed diagnostic information about the participants (n=8) diagnosed with potential celiac disease.**

| Participant | DGP-IgG  U/ml 2008 | TG2-IgA U/ml 2008 | TG2-IgA U/ml 2013^a^ | Marsh-Oberhuber classification | IEL/100 epithelial cells | % TCR γ/δ+ CD3+ IEL | TCRγ/δ+  CD3+/100 epithelial cells |
| --- | --- | --- | --- | --- | --- | --- | --- |
| 1 | 18 | 12 | 0.9 | 0 | 24 | 33 | 8 |
| 2 | 7.6 | 4.1 | 11 | 1 | 38 | 41 | 16 |
| 3 | 13 | 6.7 | 5 | 1 | 27 | 28 | 8 |
| 4 | 12 | 4.5 | 7 | 0 | 21 | 29 | 6 |
| 5^b^ | 7.2 | 17 | 0.6 | 0 | 22 | 46 | 10 |
| 6 | 6.2 | 25 | 11 | 1 | 37 | 39 | 14 |
| 7 | 48 | 82 | 73 | 1 | 37 | 24 | 9 |
| 8 | 10 | 21 | 2 | 1 | 34 | 22 | 7 |

^a^Most patients had reduced serum TG2-IgA levels between inclusion (2008) and time of biopsy (2013), suggesting reduced gluten intake or, alternatively, reduced immune response as illustrated in participant 5^b^ , who was diagnosed with autoimmune hepatitis in 2011 and treated with prednisolone (variable dosage) and low-dose methotrexate for immune suppression, which may have reduced the immune reaction to gliadin.

**Supplementary material S2**

**Different methods for estimating the prevalence of celiac disease (CD) in unbiopsied patients**

Different methods were used to estimate the prevalence of CD in the unbiopsied participants and the prevalence in the population. If the prevalence of CD in the biopsied participants (49%) is used to estimate the prevalence in the serum positive but unbiopsied participants (n=103), then 49% of the 103 unbiopsied participants may have had CD (n=50). This simple estimation could be prone to selection bias because individuals with more symptoms and a higher risk of having CD may more readily accept the invitation to gastroduodenoscopy.

However, the TG2-IgA titer distribution was almost identical in the two groups (see Figure 2). The prevalence may, therefore, be estimated from the serum TG2-IgA titer-associated disease prevalence in the biopsied participants. All of those with a serum TG2-IgA titer > 25 U/ml had either active (98%; n=45) or potential CD (2%; n=1); 87% of those with a TG2-IgA titer between 25 and 10 U/ml (26/30) and 73% of those with a TG2-IgA titer ≥ seven but <10 U/ml (11/15) had CD. Using this TG2-IgA titer-associated disease prevalence in the unbiopsied, TG2-IgA-positive participants, 98% of the 32 unbiopsied participants with TG2-IgA titers >25 U/ml (n=31.4), 87% of those 11 with TG2-IgA titers from 10 to 25 U/m (n=9.6) and 73.3% of those 12 with TG2-IgA titers ≥ 7 but <10 U/ml (n=8.8) would have had CD. This TG2-IgA titer-based estimation revealed a similar number of celiac patients (n=50) in the 103 TG2-IgA serum-positive, unbiopsied participants, as did the CD prevalence data in the biopsied participants (n=50). Adding 50 patients to those 84 who were diagnosed with biopsy verification, 134 of the 12 190 participants had previously undiagnosed CD (1.10%, 95% CI 0.91-1.27).

Finally, it is possible to use the fraction of the invited participants who accepted the invitation to gastroduodenoscopy. The 173 biopsied participants (Figure 1) represented 62.68% of the 276 invited participants. If the original screening population is reduced to the same fraction as those biopsied, then there were 84 patients with undiagnosed CD in a population of (12 190 *0.6268) = 7 641, and consequently, 1.1% had undiagnosed CD (and 8/7 641 (0.15%)) had potential CD).

Thus, three different estimation techniques resulted in the same prevalence of undiagnosed CD. Adding this prevalence (1.10%) to that of previously diagnosed patients (0.37% of 12 981 participants), 1.47% of the population had CD (95% CI 1.26-1.66), of which 75% was previously undiagnosed.

**Supplementary material S3**

**Diagnosing celiac disease (CD) based on serum TG2-IgA and/or DGP-IgG titers, without biopsy verification**

Increasing the TG2-IgA threshold to ≥10 U/ml excluded all participants without CD autoimmunity but unfortunately also 15 patients with CD. Thus, all patients with TG2-IgA ≥ 10 U/ml, regardless of their DGP-IgG titers, had either active (n=70; PPV=0.93) or potential (n=5) CD.

However, using a similar diagnostic approach that has been adapted for children based on serum TG2-IgA titers only, all 26 patients with TG2-IgA serum levels ≥ 10 x upper normal limit (≥70 U/ml) would have been correctly diagnosed with CD, without intestinal biopsy verification. Although none of the controls had such high TG2-IgA serum titers, one patient with potential CD (Marsh-Oberhuber-1 lesion) had similarly high levels. Thus, 96% of these participants had active CD (PPV = 0.96). Using a similar argument for serum DGP-IgG titer would include only 16 patients with more than 10 x upper normal limit (≥70 U/ml), of which 14 had celiac disease and two did not (PPV = 0.88). All 11 biopsied participants with both TG2-IgA and DGP-IgG ≥ 70 U/ml had CD (PPV = 1.0), but this number accounted for only 13% of the biopsied patients with CD.

**Supplementary material S4**

**Table 5: Additional information about the 84 patients diagnosed with celiac disease.**

| **Patient** (n=84) | **Marsh-Oberhuber scores** | **IEL/100 epithelial cells** | **DGP-IgG** U/ml | **TG2-IgA** U/ml | **HLA-DQ** |
| --- | --- | --- | --- | --- | --- |
| 1 | 3a | 33 | 58 | 34 | 2.5 |
| 2 | 3b | 64 | 37 | 128 | 2.5 |
| 3 | 3c | 64 | 4.5 | 37 | 2.5 |
| 4 | 3c | 35 | 12 | 13 | 2.5 |
| 5 | 3c | 80 | 119 | 70 | 2.5 |
| 6 | 3a | 34 | 5.6 | 9.7 | 7.5 |
| 7 | 3b | 49 | 13 | 52 | 2.5 |
| 8 | 3c | 72 | 114 | 127 | 2.5 |
| 9 | 3c | 56 | 27 | 79 | 2.5 |
| 10 | 3a | 77 | 88 | 95 | 2.5 |
| 11 | 3b | 55 | 185 | 15 | 2.5 |
| 12 | 3a | 56 | 2.6 | 13 | 2.5 |
| 13 | 3b | 52 | 28 | 107 | 2.5 |
| 14 | 3b | 53 | 9.6 | 24 | 2.5 |
| 15 | 3a | 35 | 51 | 27 | 2.5 |
| 16 | 3b | 30 | 17 | 128 | 2.5 |
| 17 | 3a | 32 | 13 | 52 | 2.5 |
| 18 | 3b | 91 | 73 | 115 | 2.5 |
| 19 | 3b | 59 | 32 | 24 | 8 |
| 20 | 3a | 44 | 23 | 35 | 8 |
| 21 | 2 | 38 | 0.8 | 7 | 2.5 |
| 22 | 3c | 61 | 20 | 48 | 2.5 |
| 23 | 3a | 70 | 38 | 128 | 2.5 |
| 24 | 3c | 64 | 5 | 18 | 2.5 |
| 25 | 3b | 48 | 32 | 12 | 2.5 |
| 26 | 3a | 27 | 2.3 | 14 | 2.5 |
| 27 | 3a | 26 | 5.9 | 10 | 2.5 |
| 28 | 3a | 42 | 39 | 109 | 2.5 |
| 29 | 3a | 23 | 11 | 14 | 2.5 |
| 30 | 3c | 51 | 171 | 96 | 8 |
| 31 | 3a | 44 | 5.8 | 45 | 8 |
| 32 | 3a | 32 | 8.2 | 8.6 | ND |
| 33 | 3b | 33 | 14 | 128 | 2.5 |
| 34 | 3c | 49 | 1.6 | 8.2 | 2.5 |

| **Patient** | **Marsh-Oberhuber scores** | **IEL/100 epithelial cells** | **DGP-IgG**  U/ml | **TG2-IgA**  U/ml | **HLA-DQ** |
| --- | --- | --- | --- | --- | --- |
| 35 | 3a | 45 | 36 | 4 | 2.5 |
| 36 | 3c | 52 | 37 | 28 | 2.5 |
| 37 | 3c | 32 | 21 | 37 | 2.5 |
| 38 | 3a | 30 | 11 | 11 | 8 |
| 39 | 3c | 50 | 6.5 | 11 | 2.5 |
| 40 | 3b | 71 | 9.5 | 8.1 | 2.2 |
| 41 | 3c | 42 | 65 | 128 | 2.5 |
| 42 | 3a | 48 | 62 | 24 | 2.5 |
| 43 | 3c | 80 | 76 | 128 | 2.5 |
| 44 | 3b | 103 | 4.3 | 15 | 2.5 |
| 45 | 3b | 75 | 111 | 128 | 2.5 |
| 46 | 3b | 141 | 2.1 | 13 | 2.5 |
| 47 | 3a | 41 | 42 | 7.6 | 2.5 |
| 48 | 3a | 75 | 9.1 | 11 | 2.5 |
| 49 | 3a | 32 | 9.3 | 11 | 2.5 |
| 50 | 3b | 84 | 7.3 | 8.9 | 8 |
| 51 | 3a | 65 | 7.3 | 73 | 2.5 |
| 52 | 3b | 61 | 3.9 | 13 | 2.5 |
| 53 | 3c | 85 | 76 | 128 | 2.5 |
| 54 | 3c | 81 | 35 | 53 | 2.5 |
| 55 | 3b | 64 | 2.8 | 31 | 2.5 |
| 56 | 3a | 31 | 135 | 44 | 2.5 |
| 57 | 3a | 34 | 2.2 | 13 | 2.5 |
| 58 | 3c | 55 | 0.1 | 128 | 2.5 |
| 59 | 3c | 61 | 96 | 117 | 2.5 |
| 60 | 3a | 29 | 9.6 | 34 | 2.2 |
| 61 | 3c | 112 | 20 | 16 | 8 |
| 62 | 3b | 43 | 3.6 | 12 | 8 |
| 63 | 3a | 50 | 9.2 | 1.4 | 2.5 |
| 64 | 3a | 40 | 4.4 | 45 | 2.5 |
| 65 | 3a | 38 | 52 | 8.4 | 2.5 |
| 66 | 3c | 82 | 82 | 128 | 2.5 |
| 67 | 3b | 57 | 17 | 128 | 2.5 |
| 68 | 3a | 27 | 1.8 | 7.7 | 2.2 |
| 69 | 3c | 64 | 9.6 | 11 | 2.5 |
| 70 | 2 | 29 | 0.5 | 12 | 2.5 |
| 71 | 3b | 53 | 32 | 17 | ND |
| 72 | 3c | 43 | 85 | 128 | 2.5 |

| **Patient** | **Marsh-Oberhuber scores** | **IEL/100 epithelial cells** | **DGP-IgG**  U/ml | **TG2-IgA**  U/ml | **HLA-DQ** |
| --- | --- | --- | --- | --- | --- |
| 73 | 3c | 36 | 17 | 31 | 2.5 |
| 74 | 3a | 63 | 12 | 8 | 2.5 |
| 75 | 3a | 37 | 5 | 31 | 2.5 |
| 76 | 3a | 38 | 8 | 1.1 | 2.5 |
| 77 | 3a | 55 | 14 | 79 | 2.5 |
| 78 | 3c | 47 | 37 | 17 | 2.5 |
| 79 | 3c | 56 | 38 | 102 | 8 |
| 80 | 3a | 47 | 50 | 38 | 2.5 |
| 81 | 3c | 61 | 4.5 | 113 | 2.5 |
| 82 | 3a | 42 | 10 | 17 | 2.5 |
| 83 | 3c | 84 | 80 | 7.9 | 2.5 |
| 84 | 2 | 62 | 8.3 | 15 | 2.5 |
